# Inflammatory Biomarkers and Risk of Schizophrenia: A 2-Sample Mendelian Randomization Study

**DOI:** 10.1001/jamapsychiatry.2017.3191

**Published:** 2017-11-01

**Authors:** Fernando Pires Hartwig, Maria Carolina Borges, Bernardo Lessa Horta, Jack Bowden, George Davey Smith

**Affiliations:** 1 Postgraduate Program in Epidemiology, Federal University of Pelotas, Pelotas, Brazil; 2 Medical Research Council Integrative Epidemiology Unit, University of Bristol, Bristol, England; 3 School of Social and Community Medicine, University of Bristol, Bristol, England

## Abstract

**Importance:**

Positive associations between inflammatory biomarkers and risk of psychiatric disorders, including schizophrenia, have been reported in observational studies. However, conventional observational studies are prone to bias, such as reverse causation and residual confounding, thus limiting our understanding of the effect (if any) of inflammatory biomarkers on schizophrenia risk.

**Objective:**

To evaluate whether inflammatory biomarkers have an effect on the risk of developing schizophrenia.

**Design, Setting, and Participants:**

Two-sample mendelian randomization study using genetic variants associated with inflammatory biomarkers as instrumental variables to improve inference. Summary association results from large consortia of candidate gene or genome-wide association studies, including several epidemiologic studies with different designs, were used. Gene-inflammatory biomarker associations were estimated in pooled samples ranging from 1645 to more than 80 000 individuals, while gene-schizophrenia associations were estimated in more than 30 000 cases and more than 45 000 ancestry-matched controls. In most studies included in the consortia, participants were of European ancestry, and the prevalence of men was approximately 50%. All studies were conducted in adults, with a wide age range (18 to 80 years).

**Exposures:**

Genetically elevated circulating levels of C-reactive protein (CRP), interleukin-1 receptor antagonist (IL-1Ra), and soluble interleukin-6 receptor (sIL-6R).

**Main Outcomes and Measures:**

Risk of developing schizophrenia. Individuals with schizophrenia or schizoaffective disorders were included as cases. Given that many studies contributed to the analyses, different diagnostic procedures were used.

**Results:**

The pooled odds ratio estimate using 18 CRP genetic instruments was 0.90 (random effects 95% CI, 0.84-0.97; *P* = .005) per 2-fold increment in CRP levels; consistent results were obtained using different mendelian randomization methods and a more conservative set of instruments. The odds ratio for sIL-6R was 1.06 (95% CI, 1.01-1.12; *P* = .02) per 2-fold increment. Estimates for IL-1Ra were inconsistent among instruments, and pooled estimates were imprecise and centered on the null.

**Conclusions and Relevance:**

Under mendelian randomization assumptions, our findings suggest a protective effect of CRP and a risk-increasing effect of sIL-6R (potentially mediated at least in part by CRP) on schizophrenia risk. It is possible that such effects are a result of increased susceptibility to early life infection.

## Key Points

**Question:**

What is the effect of increased inflammatory biomarkers on the risk of developing schizophrenia?

**Findings:**

In this 2-sample mendelian randomization study using summary gene-biomarker association results estimated in pooled samples ranging from 1645 to more than 80 000 individuals, 2-fold increments in circulating levels of C-reactive protein and soluble interleukin-1 receptor levels were associated with a 10% reduction and a 6% increase in the lifetime odds of developing schizophrenia.

**Meaning:**

We found that blockade of interleukin-6 effects and low C-reactive protein levels might increase schizophrenia risk, possibly due to increased susceptibility to early life infection.

## Teaser

This study uses mendelian randomization of candidate gene or genome-wide association studies to evaluate the association between inflammatory biomarkers and the risk of schizophrenia.

## Introduction

Neuropsychiatric disorders are major contributors to the burden of disease worldwide due to their substantial effect on disability.^1,2^ More than one-third of the top 25 global causes of disease burden in 2013, measured as years lived with disability, fall into this category of disorders.^1^ Schizophrenia ranks 11th among the leading global causes of years lived with disability,^1^ and its high burden is associated with substantial personal and societal costs.^3^

An association between schizophrenia and the immune system was suggested more than a century ago.^4^ Infections during pregnancy^5^ and early childhood,^6^ as well as autoimmune diseases,^7,8^ have been linked to an increased risk of schizophrenia. In addition, findings from the largest genome-wide association study of schizophrenia to date corroborate that immune dysregulation plays a role in the pathogenesis of schizophrenia.^9^ Inflammation has been hypothesized as a potential mechanism linking the immune response to the pathogenesis of schizophrenia and other neuropsychiatric disorders because cytokines may influence multiple neurologic processes, including neurotransmitter metabolism, neuroendocrine function, and neural plasticity.^4,10,11,12^ Observational epidemiologic studies, mainly of cross-sectional design, indicate that circulating levels of several cytokines,^13^ such as interleukin (IL)-1β and IL-6, and C-reactive protein (CRP),^14,15^ are higher in individuals with schizophrenia. Meta-analysis of randomized clinical trials suggested that anti-inflammatory drugs improve the symptoms of the syndrome, but only a few studies with small sample sizes are available.^16^

Not only are there relatively few studies investigating the association between inflammatory biomarkers and schizophrenia in humans, they are largely of observational nature. Conventional observational studies may have important limitations, such as reverse causation and residual confounding,^17,18,19^ which hamper conclusions on whether specific anti-inflammatory agents could reduce the risk of developing schizophrenia. Genetic variants can be used as instrumental variables of modifiable exposures in a mendelian randomization (MR) design to improve the inference in observational studies. Justifications to rely on MR as a more robust method for causal inference than conventional observational studies include Mendel’s laws and the fact that genotypes of germline genetic variation are defined at conception and are generally not associated with conventional confounders of observational studies.^18,19,20^

Mendelian randomization has been used to investigate the effect of circulating CRP levels on schizophrenia risk. In a large, Danish population-based study using 4 genetic instruments in the *CRP* gene region, the point estimate suggested a risk-increasing effect, but the lower limit of the 95% CIs did not allow excluding the possibility of important protective effects.^21^ Two subsequent studies, both using the same summary association data sets in a 2-sample MR design, reported directionally inconsistent estimates,^22,23^ likely due to a data harmonization error in 1 of them.^24^ Moreover, neither of the 2-sample MR studies performed substantial sensitivity analyses. 

We conducted this study to investigate the effect of inflammatory markers on schizophrenia risk in a 2-sample MR design. We used genetic variants associated with inflammatory biomarkers as instrumental variables to improve inference for a possible influence of the inflammatory biomarkers on risk of developing schizophrenia.^25^

## Methods

### Data sets

We obtained summary association results for 4 sets of genetic instruments: liberal CRP (instruments selected using solely statistical criteria^26^), conservative CRP (instruments restricted to the *CRP* gene region^27^), interleukin-1 receptor antagonist (IL-1Ra),^28^ and soluble interleukin-6 receptor (sIL-6R).^29^ Summary associations between each instrument and schizophrenia risk were obtained from the largest schizophrenia genome-wide association study to date.^9^ The eMethods in the Supplement provides a description of each data set. Briefly, summary association results from large consortia of candidate gene or genome-wide association studies were included. Gene-biomarker associations were estimated in pooled samples ranging from 1645 to more than 80 000 individuals, while gene-schizophrenia associations were estimated in more than 30 000 cases and more than 45 000 ancestry-matched controls. In most studies included in the consortia, participants were of European ancestry, and the prevalence of men was approximately 50%. All studies were conducted in adults, with a wide age range (18 to >80 years). The summary genetic associations data sets were harmonized as described elsewhere^24^ and are reported in eTable 1 and eTable 2 in the Supplement. This project used only publicly available summarized (ie, aggregated) results from published meta-analyses of genome-wide association studies. Individual- and study-level aggregated results were not used.

### Statistical Analysis

Single-nucleotide polymorphism (SNP) biomarker associations were collected in ln-transformed units. Odds ratio (OR) estimates of schizophrenia per 2-fold increments in circulating inflammatory biomarker levels were obtained as follows: 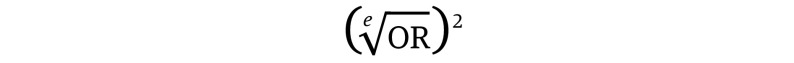
where OR is the odds ratio estimate per 1-ln increment in biomarker levels and *e* is the base of the natural logarithm.

Mendelian randomization requires that the genetic instruments are associated with the modifiable exposure of interest (assumption 1), and any association between the instruments and the outcome is mediated by the exposure (assumption 2). Given that only assumption 1 is empirically verifiable, careful consideration of potential violations of assumption 2 (due to factors such as population stratification, linkage disequilibrium, canalization, or horizontal pleiotropy) is important to minimize bias.^18^ An SNP that violates these assumptions is referred to as an invalid instrumental variable and its inclusion in MR analyses may bias the results.

Five MR methods were used.

1. Ratio method. This method was used to obtain individual SNP estimates by dividing the SNP schizophrenia by the corresponding SNP biomarker effect estimates. Standard errors were estimated using the delta method^30^ assuming the uncertainty in the SNP-exposure association estimates was negligible (the no measurement error [NOME] assumption); this method corresponds to dividing, for each SNP, the SE of SNP schizophrenia association by the absolute value of the SNP biomarker effect estimate. These SEs were then used to perform weighted analyses using methods 2 to 4.
2. Inverse variance weighting (IVW). The IVW estimate is the inverse variance weighted mean of ratio estimates from 2 or more instruments.^25^ This method assumes that all SNPs are valid instruments or are invalid in such a way that the overall bias is zero. We performed both fixed and multiplicative random effects IVW since the fixed effects method may be overprecise in the presence of heterogeneity that can occur due to, among other factors, horizontal pleiotropy or, more simply, off-target genetic effects.^31^
3. Weighted generalized linear regression. This method is similar to the IVW method but allows accounting for the correlation between the genetic instruments.^32^ The weighted generalized linear regression method was used instead of the IVW method when utilizing the conservative set of CRP genetic instruments, which comprised variants in partial linkage disequilibrium (eTable 3 in the Supplement).
4. Weighted median. The weighted median estimate is the median of the weighted empirical distribution function of individual SNP ratio estimates. It differs from a simple median estimate because the weight of each SNP in the overall estimate depends on the precision of its ratio estimate. More specifically, 50% of the weights in the analysis come from ratio estimates smaller than or equal to the weighted median. This method provides a consistent effect estimate if more than 50% of the information comes from valid SNPs.^33^
5. Mendelian randomization Egger regression. MR Egger regression consists of a weighted linear regression of SNP schizophrenia against SNP biomarker effect estimates. Assuming that horizontal pleiotropic effects and SNP exposure associations are uncorrelated (ie, the instrument strength independent of direct effects assumption), MR Egger regression provides a valid effect estimate even if all SNPs are invalid instruments. Moreover, the MR Egger intercept can be interpreted as a test of overall unbalanced horizontal pleiotropy because one would expect a null y-intercept (ie, the mean value of the SNP schizophrenia associations when the SNP biomarker association is zero) if there are no horizontal pleiotropic effects.^34^ Both fixed and multiplicative random effects versions of the MR Egger regression method were performed.

Measurement error in the SNP exposure associations (ie, NOME violation) is always present to some degree. In the 2-sample setting (which is the case in our study), NOME violation attenuates the effect estimates and also affects MR Egger regression intercept. The degree of NOME violation in IVW and MR Egger regression, respectively, can be quantified by the following statistics^35^:
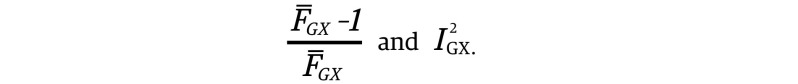
Both range from 0% to 100% and can be interpreted as the amount of attenuation in the effect estimates due to NOME violations.^35,36^ Such violations can be accounted for using the simulation extrapolation method, which was applied to MR Egger regression.^36^

The Cochran *Q* test for heterogeneity was applied to the liberal CRP set to test for the presence of horizontal pleiotropy. This test assumes that all valid genetic instruments estimate the same effect.^37^ Moreover, to identify potentially influential instruments in the liberal CRP set of instruments, we applied a range of influence tests (described in detail in the eMethods in the Supplement).^38,39^ Tests of influence were complemented by a leave-1-out approach to evaluate the influence of each SNP.

We used available data on the association between the sIL-6R genetic instrument and CRP levels^29^ in a mediation analysis evaluating the potential mediating effect of CRP in the association between sIL-6R levels and schizophrenia risk. We used MR to obtain effect estimates of the exposure-outcome (ie, sIL-6R levels and schizophrenia risk), exposure-mediator (ie, sIL-6R levels and CRP levels), and mediator-outcome (ie, CRP levels and schizophrenia risk) associations. The exposure-mediator and mediator-outcome associations can be used to estimate the expected effect of sIL-6R levels on schizophrenia risk assuming that CRP levels fully mediate this association. This effect estimate can then be contrasted to the observed exposure-outcome association to gain insights into the mediating effect of the putative mediator (the calculation is described in detail in the eMethods in the Supplement).^40^

Analyses were performed using R, version 3.2.4 (http://www.r-project.org).

## Results

Table 1 displays the association between genetically elevated inflammatory biomarkers and schizophrenia. Regarding CRP, when the liberal set of 18 CRP-associated variants was used, results were consistent among the 3 MR methods, with ORs of schizophrenia of 0.90 (random effects 95% CI, 0.84-0.97), 0.91 (95% CI, 0.85-0.98), and 0.93 (random effects 95% CI, 0.82-1.05) per 2-fold increment in circulating CRP levels using IVW, weighted median, and MR Egger regression approaches, respectively (Figure). The following statistical results:
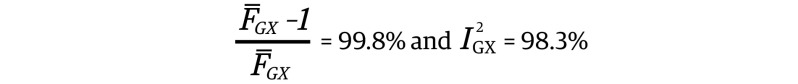
suggest that measurement errors in the SNP CRP associations were not substantially attenuating the effect estimates. Regular and simulation extrapolation–corrected MR Egger regression results were virtually identical, so only the first result was shown.

**Table 1.** Odds and Odds Ratios of Schizophrenia per 2-Fold Increments in Inflammatory Markers Based on MR

SNP Set and MR Method	Parameter	OR or Odds (95% CI)	*P* Value
CRP, liberal (n = 18)^a^			
IVW (FE)	OR	0.90 (0.86-0.95)	<.001
IVW (RE)	OR	0.90 (0.84-0.97)	.005
MR Egger (FE)	Intercept (odds)	1.00 (0.99-1.00)	.42
	OR	0.93 (0.85-1.01)	.09
MR Egger (RE)	Intercept (odds)	1.00 (0.99-1.01)	.57
	OR	0.93 (0.82-1.05)	.21
Weighted median	OR	0.91 (0.85-0.98)	.02
CRP, conservative (n = 4)^b^			
Ratio: rs1130864	OR	0.92 (0.81-1.05)	.21
Ratio: rs1205	OR	0.92 (0.84-1.01)	.06
Ratio: rs1800947	OR	0.90 (0.79-1.02)	.10
Ratio: rs3093077	OR	0.97 (0.84-1.12)	.69
WGLR	OR	0.93 (0.86-1.00)	.04
IL-1Ra (n = 2)			
Ratio: rs1542176	OR	1.02 (0.98-1.07)	.35
Ratio: rs6743376	OR	0.98 (0.94-1.01)	.20
IVW (FE)	OR	0.99 (0.83-1.19)	.73
IVW (RE)	OR	0.99 (0.76-1.30)	.82
sIL-6R (n = 1)			
Ratio: rs222814	OR	1.06 (1.01-1.12)	.02

Abbreviations: CRP, C-reactive protein; FE, fixed effects; IL-1Ra, interleukin-1 receptor antagonist; IVW, inverse variance weighting; MR, mendelian randomization; OR, odds ratio; RE, random effects; sIL-6R, soluble interleukin-6 receptor; SNP, single nucleotide polymorphism; WGLR, weighted generalized linear regression.

^a^ The liberal CRP set corresponds to the SNPs identified by Dehghan and colleagues.^26^

^b^ The conservative CRP set corresponds to the SNPs analyzed by Wensley and colleagues.^27^

**Figure.**
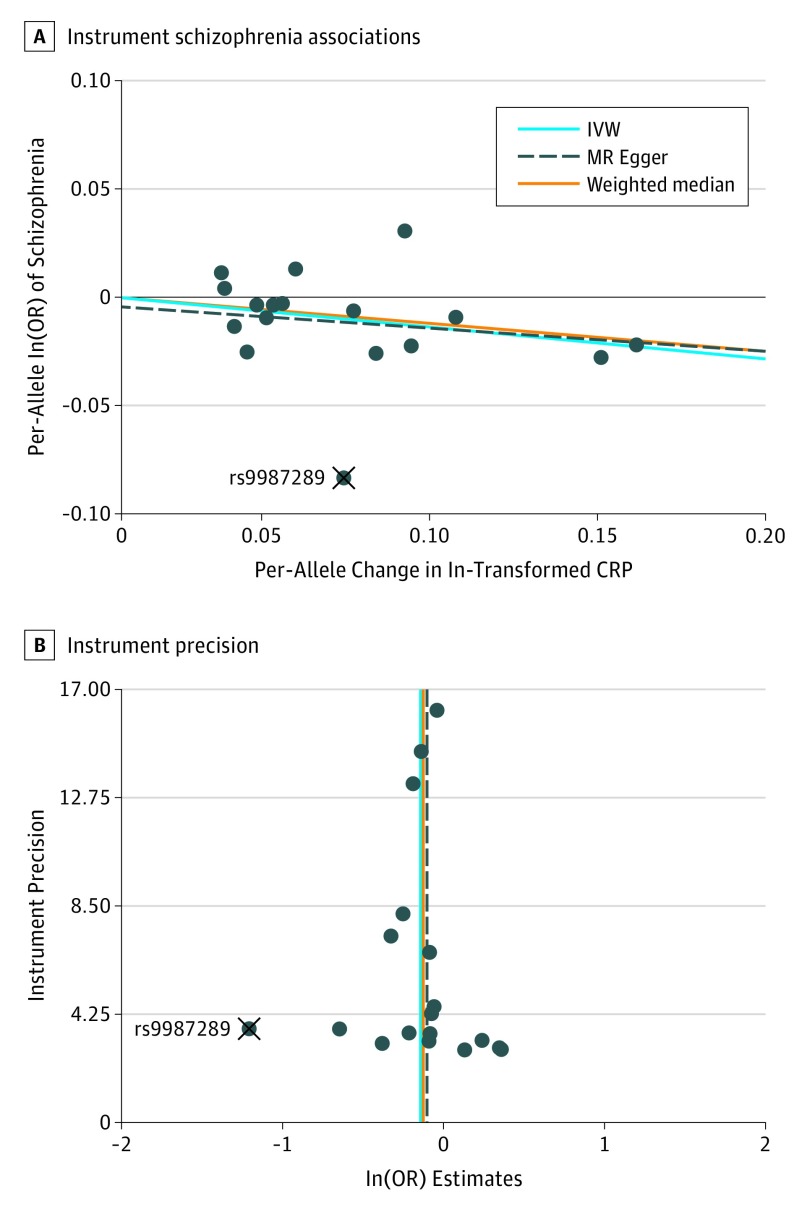
Mendelian Randomization (MR) Analyses Regarding the Effect of C-Reactive Protein (CRP) Levels on Schizophrenia Using the Liberal Set of 18 Genetic Instruments A, Instrument schizophrenia associations (y-axis) against instrument CRP associations (x-axis). B, Instrument precision (ie, instrument CRP regression coefficients divided by the correspondent instrument schizophrenia SEs) (y-axis) against individual instrument ratio estimates in log odds ratio of schizophrenia (x-axis). In(OR) indicates odds ratio estimate per 1-ln increment in biomarker levels; IVW, inverse variance weighting; X, the single genetic instrument classified as influential (rs9987289).

The MR Egger intercepts were (when rounding to 2 decimal digits) equal to 1.00 with narrow 95% CIs, suggesting no strong unbalanced horizontal pleiotropy. However, the Cochran *Q* statistic was 31.9, with an associated *P* value of .02, suggesting some heterogeneity in the effect estimates, possibly due to horizontal pleiotropy.

The results obtained using the conservative CRP set were consistent with the liberal set. Odds ratio estimates of schizophrenia per 2-fold increment in circulating CRP levels based on the ratio method ranged from 0.90 (95% CI, 0.79-1.02) to 0.97 (95% CI, 0.84-1.12). The pooled OR estimate was 0.93 (95% CI, 0.82-1.05). The 2 OR estimates for IL-1Ra were directionally inconsistent (1.02 [95% CI, 0.98-1.07] and 0.98 [95% CI, 0.94-1.01]), with a pooled IVW estimate of 0.99 (random effects 95% CI, 0.76-1.30). Finally, the OR of schizophrenia per 2-fold increments in circulating sIL-6R levels was 1.06 (95% CI, 1.01-1.12).

In the leave-1-out MR analyses using the liberal CRP set, all OR estimates of schizophrenia per 2-fold increment in circulating CRP levels were directionally consistent (Table 2). Inverse variance weighting, weighted median, and MR Egger regression estimates ranged from 0.88 to 0.91, 0.89 to 0.94, and 0.87 to 0.94, respectively. Conventional statistical significance levels were achieved in all IVW estimates and in 16 weighted median estimates but in none of the MR Egger regression estimates. The SNP rs9987289 was the only variant classified as potentially influential, and its removal had virtually no effect on the results. This single variant accounted for most of the heterogeneity in the individual instrument ratio estimates (eTable 4 in the Supplement). Therefore, any possible horizontal pleiotropy suggested by the Cochran *Q* test does not explain our findings.

**Table 2.** Odds Ratios of Schizophrenia per 2-Fold Increments in CRP Levels Based on MR Using the Liberal Set of 18 CRP-Associated Variants in a Leave-1-Out Approach

Excluded SNP	IVW (RE)		MR Egger (RE)		Weighted Median	
	OR (95% CI)	*P* Value	OR (95% CI)	*P* Value	OR (95% CI)	*P* Value
rs10521222	0.90 (0.84-0.97)	.007	0.93 (0.82-1.05)	.23	0.91 (0.85-0.97)	.02
rs10745954	0.91 (0.85-0.97)	.007	0.91 (0.81-1.04)	.15	0.91 (0.86-0.98)	.02
rs1183910	0.91 (0.84-0.98)	.02	0.94 (0.82-1.08)	.37	0.94 (0.87-1.01)	.10
rs12037222	0.90 (0.84-0.97)	.008	0.93 (0.81-1.06)	.23	0.91 (0.86-0.98)	.02
rs12239046	0.90 (0.84-0.97)	.007	0.93 (0.82-1.06)	.25	0.91 (0.85-0.97)	.02
rs1260326	0.90 (0.84-0.97)	.007	0.93 (0.82-1.05)	.23	0.91 (0.85-0.97)	.01
rs13233571	0.90 (0.84-0.96)	.005	0.93 (0.82-1.05)	.24	0.91 (0.85-0.97)	.02
rs1800961	0.90 (0.84-0.96)	.005	0.93 (0.82-1.05)	.21	0.91 (0.85-0.98)	.02
rs2794520	0.90 (0.83-0.98)	.01	0.93 (0.80-1.08)	.30	0.94 (0.87-1.01)	.13
rs2847281	0.90 (0.84-0.96)	.005	0.94 (0.83-1.07)	.35	0.91 (0.85-0.97)	.02
rs340029	0.90 (0.84-0.97)	.006	0.94 (0.82-1.07)	.30	0.91 (0.85-0.97)	.01
rs4129267	0.91 (0.85-0.98)	.01	0.93 (0.82-1.05)	.23	0.92 (0.86-0.99)	.03
rs4420065	0.91 (0.84-0.98)	.01	0.93 (0.82-1.06)	.25	0.92 (0.86-0.99)	.04
rs4420638	0.88 (0.81-0.95)	.002	0.87 (0.74-1.02)	.08	0.89 (0.83-0.95)	.004
rs4705952	0.90 (0.84-0.97)	.007	0.93 (0.82-1.06)	.25	0.91 (0.85-0.97)	.02
rs6734238	0.90 (0.84-0.97)	.007	0.93 (0.82-1.06)	.25	0.91 (0.85-0.97)	.01
rs6901250	0.90 (0.84-0.97)	.008	0.92 (0.81-1.05)	.21	0.91 (0.85-0.98)	.02
rs9987289^a^	0.91 (0.87-0.96)	.002	0.92 (0.84-1.01)	.09	0.91 (0.86-0.98)	.02

Abbreviations: CRP, C-reactive protein; IVW, inverse variance weighting; MR, mendelian randomization; OR, odds ratio; RE, random effects; SNP, single nucleotide polymorphism.

^a^ This variant was classified as potentially influential both in IVW and MR Egger.

Using the ratio method, the effect of a 1-unit increase in ln(sIL-6R) levels (used as a proxy for inhibition of IL-6 classic signaling) on ln(CRP) levels was −0.26 (95% CI, −0.32 to −0.21), and the effect of a 1-unit increase in ln(CRP) levels on the ln(OR) of schizophrenia was −0.14 (95% CI, −0.23 to −0.05). Multiplying these estimates yielded an OR of schizophrenia of 1.03 (95% CI, 1.01 to 1.04) per 2-fold increment in sIL-6R levels. The estimated proportion of the effect of inhibiting IL-6 classic signaling on schizophrenia risk that is mediated by CRP (using the IVW estimate from the liberal CRP set as the effect estimate of CRP on schizophrenia risk) was 43.8% (95% CI, 3.3% to 84.2%).

## Discussion

We used 2-sample MR to evaluate the association between inflammatory biomarkers and schizophrenia. Overall, we did not find strong evidence that lifelong exposure to increased action of these proinflammatory cytokines increases schizophrenia risk, as previously hypothesized, and, conversely, found that blockade of IL-6 effects and low CRP levels might instead increase schizophrenia risk. Moreover, part of the association between IL-6 signaling and schizophrenia might be mediated by CRP levels, which is consistent with previous knowledge on the major role of IL-6 in inducing acute-phase response and the fact that lower CRP levels are a downstream effect of inhibiting IL-6 classic signaling.^26,41,42^ For IL-1Ra, point estimates were inconsistent between instruments, and 95% CIs were large. Our study extends previous MR findings by evaluating different inflammatory markers and applying a range of sensitivity analyses.

Interleukin-6 is known for its pivotal role in integrating immune response, such as by inducing hepatic acute-phase proteins, differentiation of T cells, and tissue regeneration.^43^ Apart from being a sensitive marker of systemic inflammation and tissue damage, CRP is an acute-phase protein that contributes to host defense against infection.^44^ C-reactive protein binds to phosphocholine expressed on the surface of cells and some bacteria, which activates the complement system, promoting phagocytosis and clearance of necrotic and apoptotic cells and bacteria.^44,45^

Mechanisms underlying the association of blockade of IL-6 classic signaling and lower CRP levels with increased risk of schizophrenia are unknown. We speculate that they relate to increased susceptibility to early life infection. Blockade of IL-6 classic signaling leads to increased susceptibility to infections in mice^46^ and humans.^43^ Observational studies indicate that low levels of some acute-phase proteins in newborns were related to higher schizophrenia risk^47^ and that neonates who develop schizophrenia later in life have an impaired capacity of increasing levels of acute-phase proteins, such as CRP, in response to some maternal infections compared with controls.^48^ In adults, prospective studies indicate that higher CRP levels are related to increased susceptibility to infections.^49,50^ However, these findings should be interpreted cautiously as higher CRP levels may reflect subclinical infection, chronic activation of the inflammatory response, pre-existing disease, and socioeconomic or lifestyle characteristics. As an example, several types of infection (ie, HIV as well as hepatitis A, B, and C) appear to be more prevalent among individuals with mental disorders, including schizophrenia, in multiple settings.^51^ An MR study reported that genetic predisposition to higher CRP levels was not associated with increased infection risk in adults.^50^ To our knowledge, no existing MR study has investigated IL-6 and CRP effects on early life infection risk.

Causal inference from MR relies on some assumptions, 1 of them being that the exposure completely mediates any association between the instruments and the outcome. Most of the genetic instruments that we used have biological justifications for their selection, except for the liberal CRP set of 18 variants. Half of the variants in the liberal CRP set have been associated with 1 or more of 10 tested cardiometabolic phenotypes, while the other variants were not. Among the first half of the variants, 6 (or other variants indexing the same locus) were associated with CRP levels independently of their association with cardiometabolic phenotypes, which was not the case for the remaining 3 variants.^42^ Although these findings suggest that the CRP-associated SNPs in the liberal set are pleiotropic, our results based on these variants were consistent among the 3 MR methods (which have different assumptions regarding horizontal pleiotropy) and were corroborated by the results for the conservative CRP set and the leave-1-out analysis. The latter is also important because the liberal CRP set included the IL-6R variant rs4129267, which was not used as a genetic instrument for IL-6 classic signaling but might nevertheless influence it. Moreover, previous studies using the conservative CRP set, the IL-R1a, and the IL-6R instruments observed that, in general, those variants are not associated with conventional confounders.^27,29,52^

The SNP biomarker and SNP schizophrenia estimates were obtained in mostly European studies, thus minimizing the possibility of population stratification bias. This similarity also increases the plausibility of the 2-sample MR assumption that summary genetic association results were obtained in samples from the same or comparable populations. Regarding power, although some SNP biomarker associations were estimated in small samples, the statistical evidence for association in such data sets was generally strong. Moreover, power in the 2-sample setting depends more on the precision of the SNP outcome than on the SNP exposure association,^25^ and SNP schizophrenia associations were estimated in approximately 80 000 individuals.

### Limitations

Interpreting the magnitude of estimates for the effect of CRP and IL-6 on schizophrenia risk, as well as for the mediated effect of IL-6 by circulating CRP, requires caution. Our MR analysis likely reflects lifelong exposure to elevated cytokine and CRP levels. However, it is possible that only exposure to IL-6 and CRP in a specific window of time (eg, early life) affects schizophrenia risk. We obtained estimates for the SNP-cytokines and SNP-CRP associations from adults, but these associations might differ in early life. In addition, we used estimates for the effect of the IL-6R genetic instrument on sIL-6R as a proxy to investigate the total and the indirect (mediated by CRP) effect of blocking IL-6 classic signaling. However, this genetic instrument affects IL-6 classic signaling by increasing cleavage of membrane-bound IL-6R, which results in lower availability of membrane-bound IL-6R and higher availability of sIL-6R. Both mechanisms are likely to contribute to inhibiting IL-6 classic signaling.^43^ Finally, it is possible that IL-6 and CRP effects on schizophrenia risk are related to a maternal effect (eg, maternal susceptibility to infections during pregnancy), so that our findings are explained by the correlation between maternal and offspring genotypes. Because maternal and offspring genotypes are correlated, any effect of intrauterine exposures or maternal behavior influenced by the mother’s genetic background would also result in an association between the offspring’s genotype and risk of schizophrenia.^53^

## Conclusions

Our findings support the notion that lower CRP levels and blockade of IL-6 cell signaling—both associated with lower inflammation and acute phase response—increase schizophrenia risk. This finding suggests that the positive associations of CRP and IL-6 with schizophrenia risk in conventional observational studies are due to limitations, such as reverse causation or residual confounding. Even though our findings could be a result of horizontal pleiotropy that we failed to detect and account for, they at least suggest that increased levels of inflammatory biomarkers do not lead to substantially higher schizophrenia risk.

## Notes

Supplement. **eMethods.** Detailed Methodology **eTable 1.** SNP Biomarker and SNP Schizophrenia Associations (per Effect Allele) of the Instruments of Inflammatory Biomarkers Before Data Harmonization **eTable 2.** SNP Biomarker and SNP Schizophrenia Associations (per Effect Allele) of the Instruments of Inflammatory Biomarkers After Data Harmonization **eTable 3.** Pairwise Pearson Correlation Coefficients Among the Four CRP Genetic Instruments in the Conservative Set (Data From Phase III of the 1000 Genomes Project, Restricting to European Populations) **eTable 4.** Heterogeneity Statistics (Cochran’s Q Statistic and Associated P-Value) Associated With the Odds Ratio of Schizophrenia per Two-Fold Increments in CRP Levels Using the Liberal Set of 18 CRP-Associated Variants in a Leave-1-Out Approach.

## References

[1] Global Burden of Disease Study 2013 Collaborators Global, regional, and national incidence, prevalence, and years lived with disability for 301 acute and chronic diseases and injuries in 188 countries, 1990-2013: a systematic analysis for the Global Burden of Disease Study 2013. Lancet. 2015;386(9995):743-800. 26063472 10.1016/S0140-6736(15)60692-4 PMC4561509

[2] Whiteford HA, Ferrari AJ, Degenhardt L, Feigin V, Vos T The global burden of mental, neurological and substance use disorders: an analysis from the Global Burden of Disease Study 2010. PLoS One. 2015;10(2):e0116820. 25658103 10.1371/journal.pone.0116820 PMC4320057

[3] Knapp M, Mangalore R, Simon J The global costs of schizophrenia. Schizophr Bull. 2004;30(2):279-293. 15279046 10.1093/oxfordjournals.schbul.a007078

[4] Khandaker GM, Cousins L, Deakin J, Lennox BR, Yolken R, Jones PB Inflammation and immunity in schizophrenia: implications for pathophysiology and treatment. Lancet Psychiatry. 2015;2(3):258-270. 26359903 10.1016/S2215-0366(14)00122-9 PMC4595998

[5] Khandaker GM, Zimbron J, Lewis G, Jones PB Prenatal maternal infection, neurodevelopment and adult schizophrenia: a systematic review of population-based studies. Psychol Med. 2013;43(2):239-257. 22717193 10.1017/S0033291712000736 PMC3479084

[6] Khandaker GM, Zimbron J, Dalman C, Lewis G, Jones PB Childhood infection and adult schizophrenia: a meta-analysis of population-based studies. Schizophr Res. 2012;139(1-3):161-168. 22704639 10.1016/j.schres.2012.05.023 PMC3485564

[7] Benros ME, Nielsen PR, Nordentoft M, Eaton WW, Dalton SO, Mortensen PB Autoimmune diseases and severe infections as risk factors for schizophrenia: a 30-year population-based register study. Am J Psychiatry. 2011;168(12):1303-1310. 22193673 10.1176/appi.ajp.2011.11030516

[8] Eaton WW, Byrne M, Ewald H, Association of schizophrenia and autoimmune diseases: linkage of Danish national registers. Am J Psychiatry. 2006;163(3):521-528. 16513876 10.1176/appi.ajp.163.3.521

[9] Schizophrenia Working Group of the Psychiatric Genomics Consortium Biological insights from 108 schizophrenia-associated genetic loci. Nature. 2014;511(7510):421-427. 25056061 10.1038/nature13595 PMC4112379

[10] Dantzer R, O’Connor JC, Freund GG, Johnson RW, Kelley KW From inflammation to sickness and depression: when the immune system subjugates the brain. Nat Rev Neurosci. 2008;9(1):46-56. 18073775 10.1038/nrn2297 PMC2919277

[11] Capuron L, Miller AH Immune system to brain signaling: neuropsychopharmacological implications. Pharmacol Ther. 2011;130(2):226-238. 21334376 10.1016/j.pharmthera.2011.01.014 PMC3072299

[12] Miller AH, Maletic V, Raison CL Inflammation and its discontents: the role of cytokines in the pathophysiology of major depression. Biol Psychiatry. 2009;65(9):732-741. 19150053 10.1016/j.biopsych.2008.11.029 PMC2680424

[13] Goldsmith DR, Rapaport MH, Miller BJ A meta-analysis of blood cytokine network alterations in psychiatric patients: comparisons between schizophrenia, bipolar disorder and depression. Mol Psychiatry. 2016;21(12):1696-1709. 26903267 10.1038/mp.2016.3 PMC6056174

[14] Miller BJ, Culpepper N, Rapaport MH C-reactive protein levels in schizophrenia: a review and meta-analysis. Clin Schizophr Relat Psychoses. 2014;7(4):223-230. 23428789

[15] Fernandes BS, Steiner J, Bernstein HG, C-reactive protein is increased in schizophrenia but is not altered by antipsychotics: meta-analysis and implications. Mol Psychiatry. 2016;21(4):554-564. 26169974 10.1038/mp.2015.87

[16] Sommer IE, de Witte L, Begemann M, Kahn RS Nonsteroidal anti-inflammatory drugs in schizophrenia: ready for practice or a good start? a meta-analysis. J Clin Psychiatry. 2012;73(4):414-419. 22225599 10.4088/JCP.10r06823

[17] Phillips AN, Davey Smith G How independent are “independent” effects? relative risk estimation when correlated exposures are measured imprecisely. J Clin Epidemiol. 1991;44(11):1223-1231. 1941017 10.1016/0895-4356(91)90155-3

[18] Davey Smith G, Ebrahim S “Mendelian randomization”: can genetic epidemiology contribute to understanding environmental determinants of disease? Int J Epidemiol. 2003;32(1):1-22. 12689998 10.1093/ije/dyg070

[19] Davey Smith G Use of genetic markers and gene-diet interactions for interrogating population-level causal influences of diet on health. Genes Nutr. 2011;6(1):27-43. 21437028 10.1007/s12263-010-0181-y PMC3040803

[20] Smith GD, Lawlor DA, Harbord R, Timpson N, Day I, Ebrahim S Clustered environments and randomized genes: a fundamental distinction between conventional and genetic epidemiology. PLoS Med. 2007;4(12):e352. 18076282 10.1371/journal.pmed.0040352 PMC2121108

[21] Wium-Andersen MK, Ørsted DD, Nordestgaard BG Elevated C-reactive protein associated with late- and very-late-onset schizophrenia in the general population: a prospective study. Schizophr Bull. 2014;40(5):1117-1127. 23996346 10.1093/schbul/sbt120 PMC4133657

[22] Inoshita M, Numata S, Tajima A, A significant causal association between C-reactive protein levels and schizophrenia. Sci Rep. 2016;6:26105. 27193331 10.1038/srep26105 PMC4872134

[23] Prins BP, Abbasi A, Wong A, ; PAGE Consortium; International Stroke Genetics Consortium; Systemic Sclerosis Consortium; Treat OA Consortium; DIAGRAM Consortium; CARDIoGRAMplusC4D Consortium; ALS Consortium; International Parkinson’s Disease Genomics Consortium; Autism Spectrum Disorder Working Group of the Psychiatric Genomics Consortium; CKDGen Consortium; GERAD1 Consortium; International Consortium for Blood Pressure; Schizophrenia Working Group of the Psychiatric Genomics Consortium; Inflammation Working Group of the CHARGE Consortium Investigating the causal relationship of C-reactive protein with 32 complex somatic and psychiatric outcomes: a large-scale cross-consortium mendelian randomization study. PLoS Med. 2016;13(6):e1001976. 27327646 10.1371/journal.pmed.1001976 PMC4915710

[24] Hartwig FP, Davies NM, Hemani G, Davey Smith G Two-sample mendelian randomization: avoiding the downsides of a powerful, widely applicable but potentially fallible technique. Int J Epidemiol. 2016;45(6):1717-1726. 28338968 10.1093/ije/dyx028 PMC5722032

[25] Burgess S, Butterworth A, Thompson SG Mendelian randomization analysis with multiple genetic variants using summarized data. Genet Epidemiol. 2013;37(7):658-665. 24114802 10.1002/gepi.21758 PMC4377079

[26] Dehghan A, Dupuis J, Barbalic M, Meta-analysis of genome-wide association studies in >80 000 subjects identifies multiple loci for C-reactive protein levels. Circulation. 2011;123(7):731-738. 21300955 10.1161/CIRCULATIONAHA.110.948570 PMC3147232

[27] Wensley F, Gao P, Burgess S, ; C Reactive Protein Coronary Heart Disease Genetics Collaboration (CCGC) Association between C reactive protein and coronary heart disease: mendelian randomisation analysis based on individual participant data. BMJ. 2011;342:d548. 21325005 10.1136/bmj.d548 PMC3039696

[28] Matteini AM, Li J, Lange EM, Novel gene variants predict serum levels of the cytokines IL-18 and IL-1ra in older adults. Cytokine. 2014;65(1):10-16. 24182552 10.1016/j.cyto.2013.10.002 PMC4060632

[29] Sarwar N, Butterworth AS, Freitag DF, ; IL6R Genetics Consortium Emerging Risk Factors Collaboration Interleukin-6 receptor pathways in coronary heart disease: a collaborative meta-analysis of 82 studies. Lancet. 2012;379(9822):1205-1213. 22421339 10.1016/S0140-6736(11)61931-4 PMC3316940

[30] Thomas DC, Lawlor DA, Thompson JR Re: Estimation of bias in nongenetic observational studies using “mendelian triangulation” by Bautista et al. Ann Epidemiol. 2007;17(7):511-513. 17466535 10.1016/j.annepidem.2006.12.005

[31] Burgess S, Bowden J, Fall T, Ingelsson E, Thompson SG Sensitivity analyses for robust causal inference from mendelian randomization analyses with multiple genetic variants. Epidemiology. 2017;28(1):30-42. 27749700 10.1097/EDE.0000000000000559 PMC5133381

[32] Burgess S, Dudbridge F, Thompson SG Combining information on multiple instrumental variables in mendelian randomization: comparison of allele score and summarized data methods. Stat Med. 2016;35(11):1880-1906. 26661904 10.1002/sim.6835 PMC4832315

[33] Bowden J, Davey Smith G, Haycock PC, Burgess S Consistent estimation in mendelian randomization with some invalid instruments using a weighted median estimator. Genet Epidemiol. 2016;40(4):304-314. 27061298 10.1002/gepi.21965 PMC4849733

[34] Bowden J, Davey Smith G, Burgess S Mendelian randomization with invalid instruments: effect estimation and bias detection through Egger regression. Int J Epidemiol. 2015;44(2):512-525. 26050253 10.1093/ije/dyv080 PMC4469799

[35] Bowden J, Del Greco MF, Minelli C, Davey Smith G, Sheehan N, Thompson J A framework for the investigation of pleiotropy in two-sample summary data mendelian randomization. Stat Med. 2017;36(11):1783-1802. 28114746 10.1002/sim.7221 PMC5434863

[36] Bowden J, Del Greco MF, Minelli C, Davey Smith G, Sheehan NA, Thompson JR Assessing the suitability of summary data for two-sample mendelian randomization analyses using MR-Egger regression: the role of the I^2^ statistic. Int J Epidemiol. 2016;45(6):1961-1974. 27616674 10.1093/ije/dyw220 PMC5446088

[37] Greco MFD, Minelli C, Sheehan NA, Thompson JR Detecting pleiotropy in mendelian randomisation studies with summary data and a continuous outcome. Stat Med. 2015;34(21):2926-2940. 25950993 10.1002/sim.6522

[38] Corbin LJ, Richmond RC, Wade KH, BMI as a modifiable risk factor for type 2 diabetes: refining and understanding causal estimates using mendelian randomization. Diabetes. 2016;65(10):3002-3007. 27402723 10.2337/db16-0418 PMC5279886

[39] Hartwig FP, Bowden J, Loret de Mola C, Tovo-Rodrigues L, Davey Smith G, Horta BL Body mass index and psychiatric disorders: a mendelian randomization study. Sci Rep. 2016;6:32730. 27601421 10.1038/srep32730 PMC5013405

[40] Richmond RC, Hemani G, Tilling K, Davey Smith G, Relton CL Challenges and novel approaches for investigating molecular mediation. Hum Mol Genet. 2016;25(R2):R149-R156. 27439390 10.1093/hmg/ddw197 PMC5036871

[41] Okada Y, Takahashi A, Ohmiya H, Genome-wide association study for C-reactive protein levels identified pleiotropic associations in the IL6 locus. Hum Mol Genet. 2011;20(6):1224-1231. 21196492 10.1093/hmg/ddq551

[42] Ligthart S, de Vries PS, Uitterlinden AG, ; CHARGE Inflammation Working Group Pleiotropy among common genetic loci identified for cardiometabolic disorders and C-reactive protein. PLoS One. 2015;10(3):e0118859. 25768928 10.1371/journal.pone.0118859 PMC4358943

[43] Calabrese LH, Rose-John S IL-6 biology: implications for clinical targeting in rheumatic disease. Nat Rev Rheumatol. 2014;10(12):720-727. 25136784 10.1038/nrrheum.2014.127

[44] Pepys MB, Hirschfield GM C-reactive protein: a critical update. J Clin Invest. 2003;111(12):1805-1812. 12813013 10.1172/JCI18921 PMC161431

[45] Peisajovich A, Marnell L, Mold C, Du Clos TW C-reactive protein at the interface between innate immunity and inflammation. Expert Rev Clin Immunol. 2008;4(3):379-390. 20476927 10.1586/1744666X.4.3.379

[46] Hoge J, Yan I, Jänner N, IL-6 controls the innate immune response against *Listeria monocytogenes* via classical IL-6 signaling. J Immunol. 2013;190(2):703-711. 23241882 10.4049/jimmunol.1201044

[47] Gardner RM, Dalman C, Wicks S, Lee BK, Karlsson H Neonatal levels of acute phase proteins and later risk of non-affective psychosis. Transl Psychiatry. 2013;3:e228. 23423137 10.1038/tp.2013.5 PMC3591005

[48] Blomström Å, Gardner RM, Dalman C, Yolken RH, Karlsson H Influence of maternal infections on neonatal acute phase proteins and their interaction in the development of non-affective psychosis. Transl Psychiatry. 2015;5:e502. 25646591 10.1038/tp.2014.142 PMC4445745

[49] Kaspersen KA, Dinh KM, Erikstrup LT, Low-grade inflammation is associated with susceptibility to infection in healthy men: results from the Danish Blood Donor Study (DBDS). PLoS One. 2016;11(10):e0164220. 27701463 10.1371/journal.pone.0164220 PMC5049789

[50] Zacho J, Benfield T, Tybjærg-Hansen A, Nordestgaard BG Increased baseline C-reactive protein concentrations are associated with increased risk of infections: results from 2 large Danish population cohorts. Clin Chem. 2016;62(2):335-342. 26721294 10.1373/clinchem.2015.249680

[51] Hughes E, Bassi S, Gilbody S, Bland M, Martin F Prevalence of HIV, hepatitis B, and hepatitis C in people with severe mental illness: a systematic review and meta-analysis. Lancet Psychiatry. 2016;3(1):40-48. 26620388 10.1016/S2215-0366(15)00357-0 PMC4703902

[52] Interleukin 1 Genetics Consortium Cardiometabolic effects of genetic upregulation of the interleukin 1 receptor antagonist: a mendelian randomisation analysis. Lancet Diabetes Endocrinol. 2015;3(4):243-253. 25726324 10.1016/S2213-8587(15)00034-0 PMC4648058

[53] Lawlor D, Richmond R, Warrington N, Using mendelian randomization to determine causal effects of maternal pregnancy (intrauterine) exposures on offspring outcomes: sources of bias and methods for assessing them. Wellcome Open Res. 2017;2:11. 28405635 10.12688/wellcomeopenres.10567.1 PMC5386135

