# Supplementary material for: Inflammatory Biomarkers and Risk of Schizophrenia: A 2-Sample Mendelian Randomization Study
Source: JAMA Psychiatry. 2017 Nov 1;74(12):1226–33. doi: 10.1001/jamapsychiatry.2017.3191 (PMC6583386; doi:10.1001/jamapsychiatry.2017.3191)
Supplement: Supplement. — eMethods. Detailed Methodology eTable 1. SNP Biomarker and SNP Schizophrenia Associations (per Effect Allele) of the Instruments of Inflammatory Biomarkers Before Data Harmonization eTable 2. SNP Biomarker and SNP Schizophrenia Associations (per Effect Allele) of the Instruments of Inflammatory Biomarkers After Data Harmonization eTable 3. Pairwise Pearson Correlation Coefficients Among the Four CRP Genetic Instruments in the Conservative Set (Data From Phase III of the 1000 Genomes Project, Restricting to European Populations) eTable 4. Heterogeneity Statistics (Cochran’s Q Statistic and Associated P-Value) Associated With the Odds Ratio of Schizophrenia per Two-Fold Increments in CRP Levels Using the Liberal Set of 18 CRP-Associated Variants in a Leave-1-Out Approach. [file jamapsychiatry-74-1226-s001.pdf]

Supplementary Online Content

Hartwig FP, Borges MC, Lessa Horta B, Bowden J, Davey Smith G. Inflammatory biomarkers and risk of schizophrenia: a 2-sample mendelian randomization study. *JAMA Psychiatry*. Published online November 1, 2017. doi:10.1001/jamapsychiatry.2017.3191

eMethods. Detailed Methodology

eTable 1. SNP Biomarker and SNP Schizophrenia Associations (per Effect Allele) of the Instruments of Inflammatory Biomarkers Before Data Harmonization

eTable 2. SNP Biomarker and SNP Schizophrenia Associations (per Effect Allele) of the Instruments of Inflammatory Biomarkers After Data Harmonization

eTable 3. Pairwise Pearson Correlation Coefficients Among the Four CRP Genetic Instruments in the Conservative Set (Data From Phase III of the 1000 Genomes Project, Restricting to European Populations)

eTable 4. Heterogeneity Statistics (Cochran's Q Statistic and Associated P-Value) Associated With the Odds Ratio of Schizophrenia per Two-Fold Increments in CRP Levels Using the Liberal Set of 18 CRP-Associated Variants in a Leave-1-Out Approach

This supplementary material has been provided by the authors to give readers additional information about their work.

© 2017 Hartwig FP et al. *JAMA Psychiatry.*

eMethods. Detailed Methodology

Datasets of summary association results

The datasets of summary genetic associations datasets (shown in eTables 1 and 2) used in this work as described below.

- Circulating C reactive protein (CRP)

Two sets of CRP-associated SNPs were used. One included 18 independent SNPs identified in a GWAS in ≤82,725 European ancestry individuals.[1] SNP-CRP linear regression coefficients (changes in ln-transformed circulating CRP levels) and standard errors were obtained assuming additive genetic effects and adjusting for sex, age, recruitment site (if necessary) and relatedness (for family studies). Given that a statistical criterion was used to select these 18 instruments, they are hereafter referred to as the liberal set.

The second set included four variants in the *CRP* gene, which explain 98% of the genetic variation in this locus in European ancestry populations and have been shown to regulate CRP expression levels without changing protein sequence.[2] SNP-CRP linear regression coefficients (changes in ln-transformed circulating CRP levels) and standard errors for these variants were obtained from the CRP Coronary Heart Disease Genetics Collaboration in a sample mostly of European ancestry. The analyses were adjusted for ancestry to minimize residual confounding due to population stratification.[3] These four instruments are hereafter referred to as conservative because they are located in the *CRP* gene region and their selection incorporated biological criteria. Therefore, we considered this approach less likely to be biased by horizontal pleiotropy. These variants are in partial linkage disequilibrium with one another (eTable 3).

- Interleukin-1 receptor antagonist (IL-1Ra)

© 2017 Hartwig FP et al. *JAMA Psychiatry.*

Two SNPs located upstream of the *IL1RN* gene were selected as IL-1Ra instruments. They map to two independent loci identified in a GWAS in 4,443 European ancestry individuals [4]. C-allele carriers have higher levels of circulating IL-1Ra, an endogenous inhibitor of IL-1 downstream effects, mimicking the action of the drug Anakinra.[5]

SNP-IL-1Ra associations were estimated in standard deviation units of ln(IL-1Ra) levels in the Cardiovascular Health Study (n=3,081). These were converted to ln(IL-1Ra) units by dividing the estimates by 0.54 (the standard deviation of ln(IL-1Ra) levels in the same study).[5]

- Interleukin-6 receptor (IL-6R)

A single missense SNP (Asp358Ala) in the *IL6R* gene was selected as the IL-6R instrument. Binding of IL-6 to its membrane-bound receptor, IL-6R, is necessary for triggering classical IL-6 effects on hepatocytes and some leukocytes.[6,7] The Asp358Ala SNP increases cleave of membrane-bound IL-6R cleavage and blockade of IL-6 classical cell signaling. Therefore, downstream effects of IL-6 are attenuated in Ala-allele carriers, despite increased levels of IL-6 and soluble IL-6R (sIL-6R)[8] IL6R is a target for drugs like tocilizumab, a monoclonal antibody used to reduce systemic and articular inflammation in patients with rheumatoid arthritis.[9] A large scale Mendelian randomization study showed that this genetic variant was not associated with a wide range of biomarkers, except for those that are widely known downstream consequences of IL-6 (i.e. CRP and fibrinogen). In addition the effects of this genetic variant were consistent with the effects of tocilizumab.[10]

SNP-sIL6R associations were estimated in 1,645 individuals included in a large collaborative MR study.[11] These estimates were presented in percentage differences, which were converted to ln-transformed units.

- Schizophrenia

SNP-schizophrenia ln(odds ratio) and standard errors were downloaded from the Psychiatric Genomics Consortium website (http://www.med.unc.edu/pgc/downloads). Logistic regression assuming an additive genetic effect and adjusting for ancestry-informative principal components were performed. Analyses included 34,241 cases and 45,604 ancestry-matched controls (most of European ancestry), as well as three family-based studies of 1,235 European ancestry trios.[12]

Testing for influential genetic variants

To identify potentially influential instruments in the liberal set of CRP instruments, two tests of influence (based on studentized residuals or Cook's distance) were applied separately for IVW or MR-Egger regression.[38,39] P-values for the studentized residuals test were obtained from a Student's t-distribution with degrees of freedom equal to $L-2$ (for IVW) or $L-3$ (for MR-Egger regression), with L being the number of genetic instruments. The F distribution with joint degrees of freedom equal to $(1, L-1)$ (for IVW) or $(1, L-2)$ (for MR-Egger regression) was used for the Cook's distance test. We then applied three different statistical significance criteria to classify SNPs as potentially influential: $P<0.01$, $P<0.05$ n or $P<0.1$ in at least one of the influence tests.

© 2017 Hartwig FP et al. *JAMA Psychiatry.*

Mediation analysis

The expected effect assuming full mediation (β_E) can be estimated as $\hat{\beta}_E = \hat{\beta}_{X,M} \times \hat{\beta}_{M,Y}$, where $\hat{\beta}_{X,M}$ is the causal effect estimate of the exposure on the mediator, and $\hat{\beta}_{M,Y}$ is the causal effect estimate of the mediator on the outcome. The proportion of the effect of the exposure on the outcome that is mediated by the mediator (β_P) can then be estimated as $\hat{\beta}_P = 1 - \left(\hat{\beta}_{X;Y} - \hat{\beta}_E\right)/\hat{\beta}_{X;Y}$, where $\hat{\beta}_{X;Y}$ is the causal effect estimate of the exposure on the outcome. Standard errors for $\hat{\beta}_E$ and $\hat{\beta}_P$ were estimated using parametric bootstrap, so that in each bootstrap iteration each SNP-phenotype association was re-sampled from $N(\hat{\beta}_{a\,j}, \sigma^2_{a\,j})$, where $\hat{\beta}_{a\,j}$ is the point estimate of the effect of the jth variant on the ath phenotype, and $\sigma_{a\,j}$ is its corresponding standard error. These re-sampled summary statistics are then used to calculate $\hat{\beta}_E$ and $\hat{\beta}_P$, and this process was repeated 10,000 times, generating an empirical distribution of those statistics. Confidence intervals were then derived using the normal approximation method, using the absolute median deviation from the median (corrected for normal asymptotic consistency) of the empirical distribution as the standard error.

© 2017 Hartwig FP et al. *JAMA Psychiatry.*

eReferences

1. Dehghan A, Dupuis J, Barbalic M, et al. Meta-analysis of genome-wide association studies in >80 000 subjects identifies multiple loci for C-reactive protein levels. *Circulation.* Feb 22 2011;123(7):731-738.

2. Verzilli C, Shah T, Casas JP, et al. Bayesian meta-analysis of genetic association studies with different sets of markers. *Am J Hum Genet.* Apr 2008;82(4):859-872.

3. Wensley F, Gao P, Burgess S, et al. Association between C reactive protein and coronary heart disease: mendelian randomisation analysis based on individual participant data. *BMJ.* Feb 15 2011;342:d548.

4. Matteini AM, Li J, Lange EM, et al. Novel gene variants predict serum levels of the cytokines IL-18 and IL-1ra in older adults. *Cytokine.* Jan 2014;65(1):10-16.

5. Interleukin 1 Genetics Consortium. Cardiometabolic effects of genetic upregulation of the interleukin 1 receptor antagonist: a Mendelian randomisation analysis. *Lancet Diabetes Endocrinol.* Apr 2015;3(4):243-253.

6. Heinrich PC, Behrmann I, Haan S, Hermanns HM, Muller-Newen G, Schaper F. Principles of interleukin (IL)-6-type cytokine signalling and its regulation. *Biochem J.* Aug 15 2003;374(Pt 1):1-20.

7. Mihara M, Hashizume M, Yoshida H, Suzuki M, Shiina M. IL-6/IL-6 receptor system and its role in physiological and pathological conditions. *Clin Sci (Lond).* Feb 2012;122(4):143-159.

8. Consortium I-RMRAIRM. The interleukin-6 receptor as a target for prevention of coronary heart disease: a mendelian randomisation analysis. *Lancet.* Mar 2012;379(9822):1214-1224.

© 2017 Hartwig FP et al. *JAMA Psychiatry.*

9. Calabrese LH, Rose-John S. IL-6 biology: implications for clinical targeting in rheumatic disease. *Nat Rev Rheumatol.* Dec 2014;10(12):720-727.

10. Swerdlow DI, Holmes MV, Kuchenbaecker KB, et al. The interleukin-6 receptor as a target for prevention of coronary heart disease: a mendelian randomisation analysis. *Lancet.* Mar 31 2012;379(9822):1214-1224.

11. Sarwar N, Butterworth AS, Freitag DF, et al. Interleukin-6 receptor pathways in coronary heart disease: a collaborative meta-analysis of 82 studies. *Lancet.* Mar 31 2012;379(9822):1205-1213.

12. Schizophrenia Working Group of the Psychiatric Genomics Consortium. Biological insights from 108 schizophrenia-associated genetic loci. *Nature.* Jul 24 2014;511(7510):421-427.

© 2017 Hartwig FP et al. *JAMA Psychiatry.*

eTable 1. SNP Biomarker and SNP Schizophrenia Associations (per Effect Allele) of the Instruments of Inflammatory Biomarkers Before Data Harmonization.

SNP set	SNP	SNP-biomarker				SNP-schizophrenia			
		EA	OA	ln(levels)	SE	EA	OA	ln(OR)	SE
CRP liberal	rs2794520	C	T	0.1600	0.0060	T	C	0.0220	0.0110
CRP liberal	rs4420638	A	G	0.2360	0.0090	A	G	-0.0086	0.0146
CRP liberal	rs1183910	G	A	0.1490	0.0060	A	G	0.0278	0.0112
CRP liberal	rs4420065	C	T	0.0900	0.0050	T	C	0.0224	0.0110
CRP liberal	rs4129267	C	T	0.0790	0.0050	T	C	0.0257	0.0108
CRP liberal	rs1260326	T	C	0.0720	0.0050	T	C	-0.0061	0.0108
CRP liberal	rs12239046	C	T	0.0470	0.0060	T	C	0.0034	0.0110
CRP liberal	rs6734238	G	A	0.0500	0.0060	A	G	0.0027	0.0110
CRP liberal	rs9987289	G	A	0.0690	0.0110	A	G	0.0832	0.0188
CRP liberal	rs10745954	A	G	0.0390	0.0060	A	G	-0.0251	0.0106
CRP liberal	rs1800961	C	T	0.0880	0.0150	T	C	-0.0307	0.0300
CRP liberal	rs340029	T	C	0.0320	0.0060	T	C	0.0043	0.0112
CRP liberal	rs10521222	C	T	0.1040	0.0150	T	C	0.0091	0.0325
CRP liberal	rs12037222	A	G	0.0450	0.0070	A	G	-0.0095	0.0128
CRP liberal	rs13233571	C	T	0.0540	0.0090	T	C	-0.0131	0.0167
CRP liberal	rs2847281	A	G	0.0310	0.0060	A	G	0.0113	0.0108
CRP liberal	rs6901250	A	G	0.0350	0.0060	A	G	-0.0133	0.0113
CRP liberal	rs4705952	G	A	0.0420	0.0070	A	G	0.0033	0.0121
CRP conservative	rs3093077	C	A	0.2100	0.0179	A	C	0.0083	0.0211
CRP conservative	rs1205	C	T	0.1800	0.0102	T	C	0.0203	0.0110
CRP conservative	rs1130864	A	G	0.1300	0.0077	A	G	-0.0145	0.0116
CRP conservative	rs1800947	C	G	0.2600	0.0153	C	G	-0.0381	0.0231
IL-1Ra	rs1542176	C	T	0.3333	0.0472	T	C	-0.0099	0.0105
IL-1Ra	rs6743376	C	A	0.4630	0.0472	A	C	0.0142	0.0111
IL-6R	rs2228145	C	A	0.2949	0.0148	A	C	-0.0251	0.0108

SNP: single nucleotide polymorphism. CRP: C reactive protein. IL-1Ra: Interleukin-1 receptor antagonist. Interleukin-6 receptor. EA: effect allele. OA: other allele. OR: odds ratio.

© 2017 Hartwig FP et al. *JAMA Psychiatry.*

eTable 2. SNP Biomarker and SNP Schizophrenia Associations (per Effect Allele) of the Instruments of Inflammatory Biomarkers After Data Harmonization

SNP set	SNP	SNP-biomarker				SNP-schizophrenia			
		EA	OA	ln(levels)	SE	EA	OA	ln(OR)	SE
CRP liberal	rs2794520	C	T	0.1600	0.0060	C	T	-0.0220	0.0110
CRP liberal	rs4420638	A	G	0.2360	0.0090	A	G	-0.0086	0.0146
CRP liberal	rs1183910	G	A	0.1490	0.0060	G	A	-0.0278	0.0112
CRP liberal	rs4420065	C	T	0.0900	0.0050	C	T	-0.0224	0.0110
CRP liberal	rs4129267	C	T	0.0790	0.0050	C	T	-0.0257	0.0108
CRP liberal	rs1260326	T	C	0.0720	0.0050	T	C	-0.0061	0.0108
CRP liberal	rs12239046	C	T	0.0470	0.0060	C	T	-0.0034	0.0110
CRP liberal	rs6734238	G	A	0.0500	0.0060	G	A	-0.0027	0.0110
CRP liberal	rs9987289	G	A	0.0690	0.0110	G	A	-0.0832	0.0188
CRP liberal	rs10745954	A	G	0.0390	0.0060	A	G	-0.0251	0.0106
CRP liberal	rs1800961	C	T	0.0880	0.0150	C	T	0.0307	0.0300
CRP liberal	rs340029	T	C	0.0320	0.0060	T	C	0.0043	0.0112
CRP liberal	rs10521222	C	T	0.1040	0.0150	C	T	-0.0091	0.0325
CRP liberal	rs12037222	A	G	0.0450	0.0070	A	G	-0.0095	0.0128
CRP liberal	rs13233571	C	T	0.0540	0.0090	C	T	0.0131	0.0167
CRP liberal	rs2847281	A	G	0.0310	0.0060	A	G	0.0113	0.0108
CRP liberal	rs6901250	A	G	0.0350	0.0060	A	G	-0.0133	0.0113
CRP liberal	rs4705952	G	A	0.0420	0.0070	G	A	-0.0033	0.0121
CRP conservative	rs3093077	C	A	0.2100	0.0179	C	A	-0.0083	0.0211
CRP conservative	rs1205	C	T	0.1800	0.0102	C	T	-0.0203	0.0110
CRP conservative	rs1130864	A	G	0.1300	0.0077	A	G	-0.0145	0.0116
CRP conservative	rs1800947	C	G	0.2600	0.0153	C	G	-0.0381	0.0231
IL-1Ra	rs1542176	C	T	0.3333	0.0472	C	T	0.0099	0.0105
IL-1Ra	rs6743376	C	A	0.4630	0.0472	C	A	-0.0142	0.0111
IL-6R	rs2228145	C	A	0.2949	0.0148	C	A	0.0251	0.0108

SNP: single nucleotide polymorphism. CRP: C reactive protein. IL-1Ra: Interleukin-1 receptor antagonist. Interleukin-6 receptor. EA: effect allele. OA: other allele. OR: odds ratio.

© 2017 Hartwig FP et al. *JAMA Psychiatry.*

eTable 3. Pairwise Pearson Correlation Coefficients[a] Among the Four CRP Genetic Instruments in the Conservative Set (Data From Phase III of the 1000 Genomes Project, Restricting to European Populations)

	**rs1130864**	**rs1205**	**rs1800947**	**rs3093077**
**rs1130864**	1.000	0.453	0.133	-0.185
**rs1205**	0.453	1.000	0.326	0.196
**rs1800947**	0.133	0.326	1.000	0.088
**rs3093077**	-0.185	0.196	0.088	1.000

CRP: C reactive protein.
[a]The r^2 metric of linkage disequilibrium can be obtained by squaring the correlation coefficients.

© 2017 Hartwig FP et al. *JAMA Psychiatry.*

eTable 4. Heterogeneity Statistics (Cochran's Q Statistic and Associated P-Value) Associated With the Odds Ratio of Schizophrenia per Two-Fold Increments in CRP Levels Using the Liberal Set of 18 CRP-Associated Variants in a Leave-1-Out Approach.

**Excluded SNP**	**Q statistic**	***P***
rs10521222	31.9	0.01
rs10745954	28.5	0.03
rs1183910	31.6	0.01
rs12037222	31.9	0.01
rs12239046	31.8	0.01
rs1260326	31.8	0.01
rs13233571	30.4	0.02
rs1800961	29.8	0.02
rs2794520	31.9	0.01
rs2847281	29.8	0.02
rs340029	31.3	0.01
rs4129267	30.1	0.02
rs4420065	31.1	0.01
rs4420638	29.0	0.02
rs4705952	31.9	0.01
rs6734238	31.8	0.01
rs6901250	31.4	0.01
rs9987289[a]	16.7	0.41

SNP: single nucleotide polymorphism. CRP: C reactive protein.
[a]This variant was classified as potentially influential both in IVW and MR-Egger.

© 2017 Hartwig FP et al. *JAMA Psychiatry.*
